# Hearing loss in patients with mucopolysaccharidoses‐1 and ‐6 after hematopoietic cell transplantation: A longitudinal analysis

**DOI:** 10.1002/jimd.12277

**Published:** 2020-07-09

**Authors:** Brigitte T. A. van den Broek, Adriana L. Smit, Jaap Jan Boelens, Peter M. van Hasselt

**Affiliations:** ^1^ Sylvia Toth Center for Multidisciplinary Follow up after Hematopoietic Cell Transplantation Wilhelmina Children's Hospital, University Medical Center Utrecht, Utrecht University Utrecht The Netherlands; ^2^ Pediatric Blood and Marrow Transplantation Program, Department of Child Health Wilhelmina Children's Hospital, University Medical Center Utrecht, Utrecht University Utrecht The Netherlands; ^3^ Section of Metabolic Diseases, Department of Child Health Wilhelmina Children's Hospital, University Medical Center Utrecht, Utrecht University Utrecht The Netherlands; ^4^ Department of Otorhinolaryngology—Head and Neck Surgery University Medical Center Utrecht, Utrecht University Utrecht The Netherlands; ^5^ Brain Center Rudolf Magnus University Medical Center Utrecht, Utrecht University Utrecht The Netherlands; ^6^ Pediatric Blood and Marrow Transplantation Program, Memorial Sloan Kettering Cancer Center New York New York USA

**Keywords:** disease progression, hearing, hematopoietic cell transplantation, Hurler syndrome, lysosomal storage disease, mucopolysaccharidosis 1

## Abstract

Hearing loss is frequently seen in mucopolysaccharidoses (MPS) patients. Although hematopoietic cell transplantation (HCT) increases overall survival, disease progression is observed in certain tissues. This study describes the course of hearing loss (HL) over time in transplanted MPS patients. Transplanted MPS patients between 2003 and 2018 were included and received yearly audiological evaluation, including auditory brainstem response (ABR) or pure tone audiometry (PTA). Twenty‐eight MPS‐1 and four MPS‐6 patients were analyzed with a median follow‐up of 5 years (range 11 months–16 years). Air conduction threshold improved significantly over time (*P* < .001) with a PTA 1‐year post‐HCT of 50 ± 0.7 dB to 23 ± 11 dB 13 years post‐HCT. Bone conduction threshold worsened with a PTA 1 year post‐HCT of 10 ± 7 dB to 18 ± 9 dB 13 years post‐HCT (*P* = .34). The degree of HL varied from mainly mild‐severe early after HCT to normal‐mild at longer follow‐up. The type of HL consisted of mainly conductive in the first years post‐HCT in contrast to mainly sensorineural at longer follow‐up. MRIs of the cerebellopontine angle did not show abnormalities. HL is still seen in patients with MPS despite HCT and consists of a conductive type early after HCT in contrast to a sensorineural type at longer follow‐up in the majority of cases. Yearly follow‐up of HL is necessary to timely intervene, as hearing is important in the speech and language development of children and their academic achievements.

## Graphical abstract

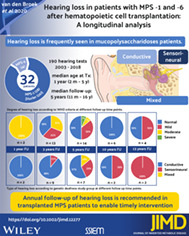

**SYNOPSIS**

Yearly follow‐up of hearing loss in transplanted MPS patients is necessary to enable timely intervention, as hearing is important in the speech and language development of children and their academic achievements.

## 1 INTRODUCTION

Hearing loss is a common feature in all subtypes of the mucopolysaccharidoses (MPS).^1^, ^2^, ^3^, ^4^, ^5^, ^6^, ^7^ The MPS are lysosomal storage disorders caused by a deficiency of one of the enzymes involving glycosaminoglycan (GAG) degradation, leading to multi‐systemic disease and, if severe, premature death.^8^ Undiagnosed MPS patients are frequently evaluated by otorhinolaryngologists because of the early‐onset of ear‐nose‐throat (ENT) manifestations, including otitis media, macroglossia, adenotonsillar hypertrophy, nasal obstruction, obstructive sleep apnea syndrome (OSAS), progressive respiratory disorders, and hearing loss.^9^

Hearing loss in MPS may be conductive, sensorineural or mixed in origin. The conductive component of hearing loss is the result of seromucinous otitis, ossicular chain deformities or disruption, or arthropathy.^10^, ^11^ Sensorineural hearing loss (SNHL) has been attributed to the accumulation of GAGs in the cochlea, auditory nerve, and brain stem.^12^

Hematopoietic cell transplantation has been used as treatment for MPS‐1, MPS‐2, MPS‐3, MPS‐4, and MPS‐6 with variable success.^13^ For MPS‐1 (Hurler syndrome), it is now standard therapy. Based on the principle of cross‐correction, HCT has dramatically increased life expectancy in MPS‐1 and attenuated symptoms in both MPS‐1 and ‐6 patients in the past decade.^14^, ^15^ In depth analyses, however, have shown disease progression in some organs but not in others despite optimized transplantation protocols.^16^, ^17^ Detailed long‐term outcome studies on hearing loss in MPS patients after HCT are scarce and most often cross‐sectional.^18^, ^19^ The extensive follow‐up program at the Sylvia Toth Center for Multidisciplinary Follow up after Hematopoietic Cell Transplantation in the Wilhelmina Children's Hospital, Utrecht, The Netherlands, has over 15 years of experience with these patients. This allows us to perform in depth longitudinal analyses and analyze the course of hearing loss in transplanted MPS patients, which are reported in this study.

## 2 METHODS

### 2.1 Data collection

All MPS‐1 and ‐6 patients treated with HCT in the Wilhelmina Children's Hospital (WKZ) of the University Medical Centre Utrecht (UMCU), The Netherlands between 2003 and 2019, were included in this study. The patients were evaluated by an experienced otorhinolaryngologist and received a hearing assessment on a yearly basis at the Sylvia Toth Center for Multidisciplinary Follow up after Hematopoietic Cell Transplantation. This study is based on retrospectively collected patient data acquired during care‐as‐usual and has been exempted for approval according to the responsible institutional committee Medical Research Involving Human Subjects Act. The MPS subtype, age at transplantation, enzyme activity level after transplantation (scored as below or above lower limit of normal, cut‐off points: 20 nmol/h/mg for alpha‐1‐iduronidase and 100 nmol/h/mg for arylsulfatase‐B), otorhinolaryngological and audiological interventions, including the insertion of a ventilation tube in the ear drum and hearing aid use were collected from the medical records.

### 2.2 Audiologic examination

Pure tone audiometry was performed repeatedly to assess hearing levels. In uncooperative patients due to a young age or cognitive impairment, auditory brainstem response (ABR) was performed during natural sleep or sedation. The hearing loss was evaluated according to the threshold in the better ear. The results of pure tone audiometry and ABR were analyzed separately.

#### 2.2.1 ABR

Click‐evoked auditory brainstem response audiometry was performed in a soundproof room during natural sleep (Synergy version 14.1; Viasys Healthcare, Surrey, UK). The ABR waveform was elicited by a click‐evoked stimulus of 0.1 ms per click, with a repetition rate of 11.7 clicks/s and alternating polarity. Electrodes were placed on both mastoids with a reference at the vertex and a ground electrode on the forehead and then band pass filtered. Response thresholds were determined by changing the stimulus level with step sizes of 10 dB down and 5 dB up method until no response was found. Experienced clinical specialists analyzed the waveform and defined the auditory brainstem response threshold in dBnHL as the lowest level at which a wave V was repeatedly present. The degree of hearing level in dB HL was agreed to be 10 dB below the estimated threshold in dBnHL as measured by this method.^20^

#### 2.2.2 Pure tone audiometry

An experienced audiology assistant performed the hearing test by air (AC) and bone conduction (BC) for the frequencies 250, 500, 1000, 2000, 4000, and 8000 Hz in a quiet room and masking as appropriate. The hearing loss levels were determined by taking the four‐frequency pure tone average (PTA) of the air conduction threshold (500‐1000‐2000‐4000 Hz).

The degree of hearing loss was categorized according to the World Health Organization (WHO) International Classification of Impairments, Disabilities, and Handicaps (WHO‐ICIDH) standard.^21^ Hearing loss was classified as normal (0‐25 dB), mild (26‐40 dB), moderate (41‐60 dB), severe (61‐80 dB), or profound (≥81 dB).

The criteria of the Genetic Deafness study group^22^ were used for the classification of the type of hearing loss. The air‐bone gap was considered normal if the average AC threshold was ≤15 dB above the BC threshold. Pure conductive hearing loss (CHL) was defined as an average BC threshold ≤20 dB with an average air‐bone gap ≥15 dB. Pure sensorineural hearing loss (SNHL) was defined as an average BC threshold >20 dB with an average air‐bone gap <15 dB. And finally, mixed hearing loss (MHL) was defined as an average BC threshold >20 dB with an average air‐bone gap of ≥15 dB.

### 2.3 Magnetic resonance imaging (MRI)

MRIs of the cerebellopontine angle were performed in a random selection of MPS‐1 patients during care‐as‐usual brain MRIs. Sagittal spin‐echo T1‐weighted images, and transverse turbo spin‐echo T2‐weighted and diffusion weighted single‐shot images were performed. Furthermore, transverse 3D T1 and T2‐weighted images were performed. The transversal 3D weighted‐image was repeated after administration of gadolinium. All images were performed on a Philips Achieva, Ingenia, or Ingenia Elition 1.5 T or 3.0 T magnetic resonance system.

### 2.4 Statistical analysis

Baseline characteristics are reported as frequencies and percentages for categorical variables and median (range) for continuous variables. The course of hearing loss over time after transplantation was analyzed with a linear mixed model. To account for nonlinearity in air conduction and the air‐bone gap, restricted cubic splines with three knots were added to the model. The four‐frequency PTA of the better ear was used as the dependent variable for AC and BC thresholds as well as for the air‐bone gap. Based on previous literature, age at time of transplantation, subtype, and enzyme level after transplantation were considered important covariates and included as fixed effects. A random intercept was included per individual to account for individual variation at baseline and a random slope (for time) for dependency across the repeated measurements within the same individual during follow‐up. To analyze the course of hearing loss for the separate frequencies, data of both ears was included. To account for dependency between both ears a random intercept per ear was included additional to the random intercept per individual. Final coefficients were estimated using restricted maximum likelihood and significance of parameters was assessed with a likelihood ratio test between the model with and without the parameter. The model assumptions including normal distributed residuals, random effects, and homogeneity of variance were confirmed visually. *P*‐values <.05 were considered significant. The R project (RStudio: Integrated Development for R. RStudio, Inc, Boston, MA) for statistical computing version 3.4.1 with the packages “lme4,” “rms,” and “ggplot2” was used for all analyses and for the creation of figures.

## 3 RESULTS

### 3.1 Patients

In total, 190 hearing observations of 32 patients were analyzed. MPS‐1 was the most frequent MPS subtype (n = 28; 88%). Within the phenotypic range of MPS‐1, Hurler syndrome was more frequent (n = 26; 93%) compared to Hurler‐Scheie (n = 2; 7%). Four patients were diagnosed with MPS‐6 (12%). The median age at transplantation for all patients was 1 year (range 2 months–5 years). Nearly all patients had leukocyte enzyme activity levels in the normal range post‐HCT (n = 30, 94%). The median follow‐up after HCT consisted of 5.0 years (range 11 months–16 years).

### 3.2 Hearing loss

#### 3.2.1 ABR

ABR was performed in 12 patients (all MPS‐1) aged between 0 and 6 years. In 6 patients (50%) ABR was performed before HCT and all these patients were <2 years. Post‐HCT ABR were performed in patients aged 2 to 6 years with a median follow‐up of 1.5 years (range 1‐3 years). The average hearing threshold determined by ABR pre‐HCT was 43 dBeHL (dB estimated hearing level). The average hearing threshold determined by ABR post‐HCT was 39 dBeHL. None of these patients had normal hearing with a threshold of <20 dB hearing loss in both ears. A conductive or mixed type hearing loss was seen in the majority of the patients (n = 10; 83%).

#### 3.2.2 Pure tone audiometry

**FIGURE 1:**
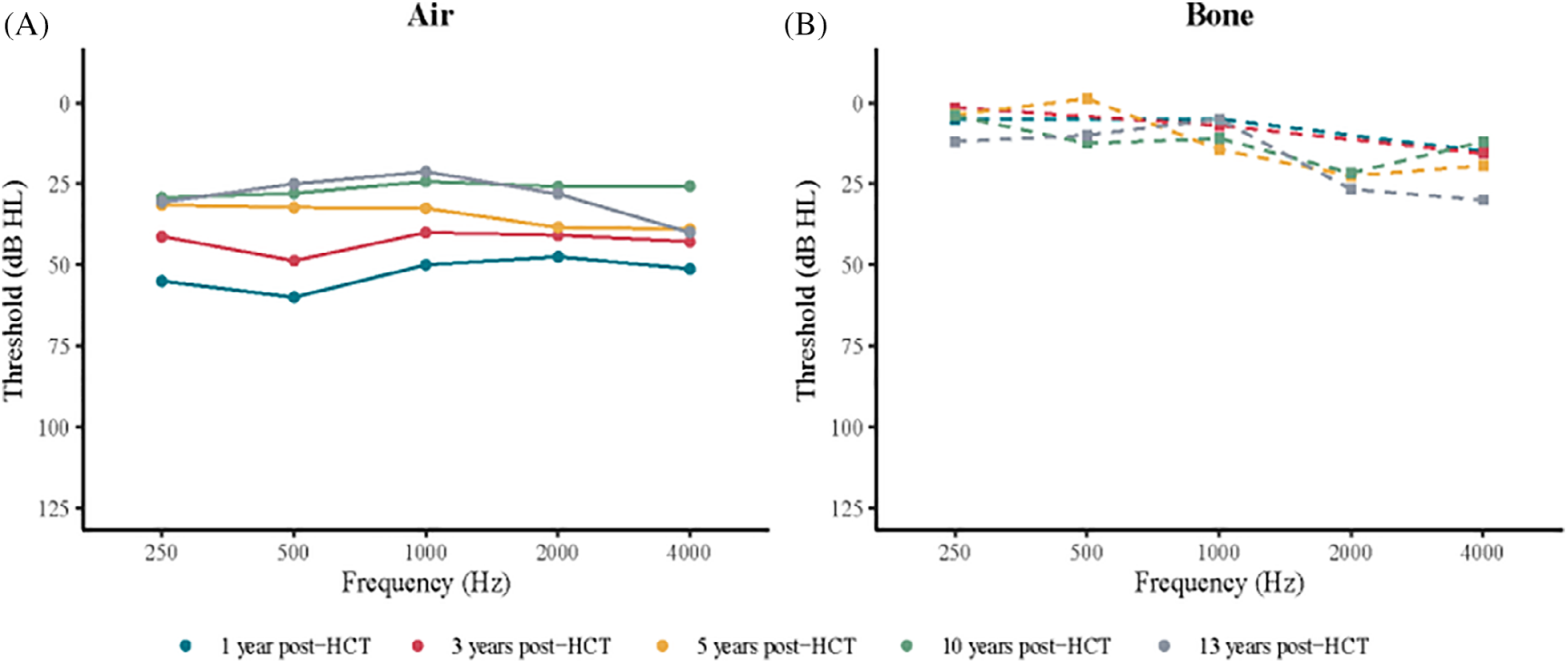
Average pure tone audiogram for air conduction, A; and bone conduction, B; at 1 year (blue), 3 years (red), 5 years (yellow), 10 years (green), and 13 years (gray) after HCT

**FIGURE 2:**
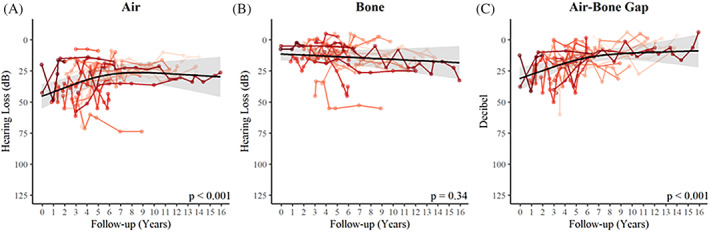
Pure tone average thresholds for air conduction, A; bone conduction, B; and the air‐bone gap, C. Air conduction, bone conduction, and air‐bone gap PTA per individual patient over time (red lines). The black line depicts the average course over time as estimated by the linear mixed model for a patient with normal enzyme activity levels after HCT and mean age at transplantation (1.22 years)

Pure tone audiometry was performed longitudinally 179 times in 22 patients from the age of 3 years and onwards (maximum age at latest follow‐up was 17 years). One year after HCT, the air conducted mean PTA was 50 dB (95% CI 49‐51 dB), after 3 years this was 34 dB (95% CI 26‐44 dB), after 5 years 30 dB (95% CI 22‐37 dB), after 10 years 24 dB (95% CI 20‐28 dB), and after 13 years 23 dB (95% CI 12‐33 dB) (Table 1; Figure 1). Over time, a significant decrease in air conduction threshold was seen (*P* < .001; Figure 2A), which correlates with improved hearing. The improvement in hearing was most profound in the first 5 years following HCT. Age at time of transplantation, enzyme activity level after HCT, and subtype did not show to be significant predictors for the outcome in this cohort (Table 2).

**TABLE 1:** Patient characteristics

Patient characteristics (n = 32)	n (%)	
MPS‐1	28 (88%)	
Hurler	26 (93%)	
Hurler‐Scheie	2 (7%)	
MPS‐6	4 (12%)	
Age at transplantation (median; range)	1 year (2 months–5 years)	
Enzyme level above LLN post‐HCT	30 (94%)	
Follow‐up after HCT (median; range)	5.0 (11 months–16 years)	
dB hearing loss at follow‐up	Air mean (95% CI)	Bone mean (95% CI)
1 year post‐HCT (n = 2)	50 (49,51)	10 (3,18)
3 years post‐HCT (n = 13)	32 (25,40)	11 (5,17)
5 years post‐HCT (n = 14)	29 (21,37)	15 (8,22)
10 years post‐HCT (n = 6)	22 (20,23)	12 (7,16)
13 years post‐HCT (n = 4)	23 (12,33)	18 (9,26)

**TABLE 2:** Linear mixed‐model estimates for air conduction threshold, bone conduction threshold, and air‐bone gap

Fixed effect		AC threshold		BC threshold		Air‐bone gap	
		Estimate (SE)	*P*	Estimate (SE)	*P*	Estimate (SE)	*P*
Intercept		45.6 (7.3)		7.46 (4.7)		30.1 (5.7)	
Follow‐up (years)		See Figure 2	**<**.**001**	See Figure 2	0.34	See Figure 2	**<**.**01**
Age at HCT		−0.0009 (0.01)	.95	0.006 (0.007)	0.34	0.001 (0.006)	.77
MPS subtype	MPS1	Reference	.49	Reference	0.09	Reference	.98
	MPS6	−7.1 (11.5)		−12.8 (8.1)		0.53 (8.2)	
Enzyme activity post‐HCT	Normal	Reference	.93	Reference	0.54	Reference	.31
	↓ Normal	−0.81 (10.6)		−3.73 (7.5)		5.42 (6.0)	

Abbreviations: AC, air conduction; BC, bone conduction; HCT, hematopoietic cell transplantation; NA, not applicable.

Bold values are indeed significant. *P* <.001 and *P* <.01.

The bone conducted mean PTA showed a tendency to increase over time but was not significant (*P* = .34; Figure 2B). When looking at the separate frequencies, the increase in bone conduction threshold was most profound in the middle range frequencies and significant in the 500 Hz frequency (*P* = .007; Figure S1). One year after HCT, the bone conducted mean PTA was 10 dB (95% CI 3‐18 dB), after 3 years it was 12 dB (95% CI 6‐17 dB), after 5 years 15 dB (95% CI 8‐21 dB), after 10 years 14 dB (95% CI 8‐20 dB), and after 13 years 18 dB (95% CI 9‐26 dB) (Table 1; Figure 1). In this cohort, bone conduction was not significantly affected by the age at transplantation, the enzyme activity level after transplantation, or subtype (Table 2).

**FIGURE 3:**
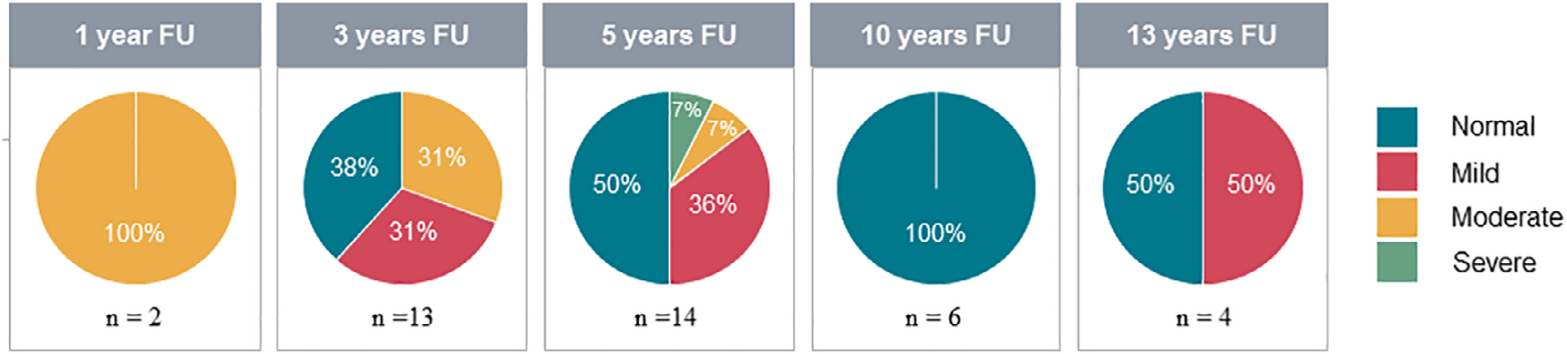
Degree of hearing loss according to WHO criteria at different follow up time points. Degree of hearing loss was based on PTA of the better ear

According to the WHO criteria defining hearing loss, 100% of patients had moderate hearing loss 1 year after HCT (n = 2). Three years after HCT, 38% had normal, 31% mild, and 31% moderate hearing loss (n = 13; Figure 3). At 5 years after HCT, 50% had normal hearing, 36% mild hearing loss, 7% moderate, and 7% severe (n = 14). Ten years after HCT, 100% had normal hearing (n = 6). Finally, at 13 years after HCT 100% had normal hearing and 50% mild hearing loss (n = 4; Figure 3). Of the patients with >10 years of follow‐up, 89% improved in the first 5 years of follow‐up. Between 5 years of follow‐up and their latest follow‐up time point, 30% worsened.

**FIGURE 4:**
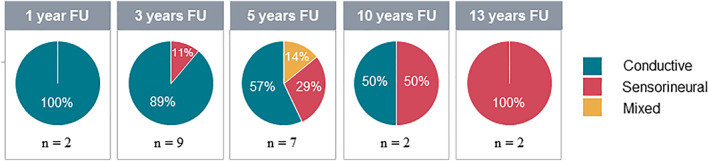
Type of hearing loss according to genetic deafness study group at different follow up time points. Type of hearing loss was based on PTA results

The type of hearing loss consisted of mainly conductive early after HCT (100% at 1 year follow‐up, 89% at 3 years follow‐up, and 57% at 5 years follow‐up; Figure 4). At later follow‐up time points the main type of hearing loss was sensorineural (50% at 10 years follow‐up, and 100% at 13 years follow‐up).

##### Hearing aids or interventions

Bilateral grommets were placed 44 times in 21 patients (66%) with a median age of 4 years (range 1‐14 years). Two patients had the MPS‐6 subtype. Fifteen patients (81%) received these ≥2 times. Of the 32 MPS patients, 12 patients were prescribed hearing amplifications (38%). All were MPS‐1 patients. The majority used bilateral hearing aids (83%), with the exception of 2 patients who received a bone conduction device (13%). The median age at the start of hearing aids use was 4 years (range 2‐15 years) at a median follow‐up of 3 years (range 1‐14 years).

### 3.3 MRI

MRIs of the cerebellopontine angle were performed in 10 patients (all MPS‐1) at median follow‐up of 12 years (range 4‐16 years). None showed any abnormalities in the n. cochlearis, n. facialis, or n. vestibularis inferior/superior, respectively. Furthermore, no abnormalities in the anatomy of the cochlea or inner ear were seen.

## 4 DISCUSSION

This longitudinal analysis, spanning more than 15 years of repeated audiological evaluation in MPS‐1 and ‐6 children after HCT at a young age, shows hearing impairment despite HCT. Although an initial improvement is seen, a significant number of patients will likely need hearing aids before reaching adulthood. The initial improvement is seen in air conduction in the first 5 years of life after HCT. Thereafter, air conduction thresholds are more or less stable with a mean hearing loss of 20 to 30 dB. Bone conduction, however, shows a slow deterioration over time which could eventually lead to the need of a hearing aid or even a cochlear implant. This knowledge is important for caregivers counseling these patients.

Detailed analysis of hearing after HCT in MPS patients was reported previously in two other studies and the results are in line with our data.^18^, ^19^ Although both were cross‐sectional studies, they did also show conductive hearing loss in the majority of early follow‐up patients vs sensorineural hearing loss at in the majority of patients with longer follow‐up. Previous studies have shown that treatment at an early age and enzyme activity levels in the normal range are important predictors for the outcome of MPS‐1 patients, including hearing.^16^, ^23^, ^24^ For the use of hearing aids, Aldenhoven et al.^16^ showed in a large study with 217 MPS‐1 patients a hazard ratio of 0.42 for patients with enzyme activity levels in the normal range compared to patients with enzyme activity level below the normal range. In our cohort however, age at transplantation and enzyme activity level post‐transplantation did not show to be predictors for hearing loss, which could be because of the small sample size or the small variation of these two variables (the mean age at transplantation was low at 1.22 years and the cohort was homogeneous with only two patients having enzyme activity levels below the normal range).

Eustachian tube dysfunction, recurrent middle ear infections and thickened middle ear mucosa by GAG deposits have been considered important factors of conductive hearing loss in MPS patients. The improvement of air conduction in the first years after HCT might be due to a combination of the natural improvement in the occurrence of chronic otitis in young children by age and a beneficial effect of HCT especially on the mucosal tissue, both resulting in a reduction of otitis episodes.^18^ Furthermore, some patients received ventilation tubes because of otitis media with effusion which will have had a positive effect on the air conduction threshold as well by reduction of the fluid related attenuation. It is important to realize that the mean PTA at 13 years follow‐up, when most children have reached early adulthood, was at the borderline between normal hearing and mild hearing loss. Due to natural deterioration of inner ear function by aging, this means the majority of patients will develop significant hearing loss in adulthood. While the air conduction threshold decreases over time, the air‐bone gap approaches 0, implying the remaining hearing loss is mostly sensorineural in origin.

The bone conducted threshold slowly increases over time especially in the middle range frequencies. This implies that hearing loss actually is progressing despite HCT. As disease progression is seen in transplanted patients especially in connective tissue (ie, skeleton and cornea),^16^, ^17^ it is reasonable to assume this is also the cause for progressive hearing loss post‐HCT. However, we cannot exclude that the hearing loss is the result of toxic effects of the (variable) HCT conditioning regimens itself or a late effect of the otitis episodes early in life. How the bone conduction threshold will develop after even longer follow‐up is uncertain, but it is likely that it continues to worsen slowly in addition to the natural deterioration of inner ear function by aging. When severe to profound SNHL is present a cochlear implant could be considered, which has already shown to improve hearing in patients with MPS‐1, 2, and 4.^25^, ^26^

The exact mechanism of SNHL in MPS patients is unknown, however Kariya et al.^27^ described the inner ear changes of 6 post‐mortem MPS‐1 patients. The organs of Corti were affected and the mean number of both inner and outer hair cells was decreased in MPS‐1 patients compared to healthy controls. Animal studies on the histopathological changes of the inner ear show similar results.^28^, ^29^ Of note, these studies were performed in untreated patients and animals. It is unknown to what extent HCT alters these changes in the inner ears of patients. The reports of successful cochlear implant performance in MPS patients support the hypothesis that SNHL pathology is limited to the inner ear and is not situated in the cochlear nerve or brain stem.^25^, ^26^ We could not report any data on inner ear pathology. However, MRIs of the cerebellopontine angle did not show any abnormalities in the anatomy, arguing against gross anatomical abnormalities of the inner ear. One study in which MRIs in untreated MPS patients were performed, did show (non‐)anatomical abnormalities in the middle and inner ear (eg, cystic cochlear apex and dilated vestibule, and middle ear effusion).^30^ Future research focusing on inner ear changes, for instance with otoacoustic emissions and advanced electrophysiological measurements could possibly help elucidate the pathophysiology of SNHL in MPS.

The MPS are rare diseases, therefore studying them inevitably results in a small number of patients, which is a limitation of this study. Unfortunately, no control data was available, which hampers to determine the specific effect of HCT on hearing. Also, because transplantation is performed in children at a very young age hearing assessments determining the exact hearing level before HCT were missing which hindered comparison pre‐ and post‐intervention. Due to the longitudinal aspect of the study, however, we were able to show the course of hearing loss over time after HCT. Finally, as this was a retrospective study, we only had data collected from care‐as‐usual.

Concluding, hearing loss is still seen after long‐term follow‐up in MPS‐1 and ‐6 children despite HCT at an early age. When looking in‐depth with a maximum follow‐up of 16 years, air conduction is initially improving after HCT. Bone conduction however is slowly worsening over time, which could be a sign of disease progression in HCT‐treated patients. To improve the quality of life of these patients, new therapies should focus on how to stop post‐transplant disease progression. Until then, MPS‐1 and ‐6 patients awaiting HCT should be informed that most likely ventilation tubes and/or hearing aids may be necessary at some point. Furthermore, after HCT, yearly and long‐term evaluation with a hearing assessment by tone audiometry and potentially otoacoustic emissions is indicated for early diagnosis of the severity and type of hearing loss and to initiate treatment of hearing loss to ensure the requirements for adequate speech and language development and academic performance in these patients.

## CONFLICT OF INTEREST

All authors state they have no competitive (financial) interests in this study.

## AUTHORSHIP CONTRIBUTIONS

Brigitte T.A. van den Broek: contributed to the design and planning and to the acquisition of the data, performed analyses, interpreted the results and drafted the manuscript including figures, and serves as guarantor for the article; Adriana L. Smit: contributed to the design and planning and to the acquisition of the data, interpret the data, and critically revised the manuscript; Jaap Jan Boelens: contributed to the acquisition of the data, interpret the data, and critically revised the manuscript; Peter M. van Hasselt: contributed to the acquisition of the data, interpret the data, and critically revised the manuscript; all authors were responsible for the medical care of the patients.

## DATA AVAILABILITY STATEMENT

The data that support the findings of this study are available from the author, PMvH, upon reasonable request.

## Supporting information


**Supplementary Figure S1** Air conduction, bone conduction and air‐bone gap per individual patient over time (blue lines) for the different threshold test frequencies (A: 250 Hz, B: 500 Hz, C: 1000 Hz, D: 2000 Hz, E: 4000 Hz). The black line depicts the average course over time for a patient with normal enzyme activity levels after HCT and mean age at transplantation (1.22 years).
