# Supplementary figures and images for: Hearing loss in patients with mucopolysaccharidoses‐1 and ‐6 after hematopoietic cell transplantation: A longitudinal analysis
Source: J Inherit Metab Dis. 2020 Jul 9;43(6):1279–87. doi: 10.1002/jimd.12277 (PMC7689745; doi:10.1002/jimd.12277)

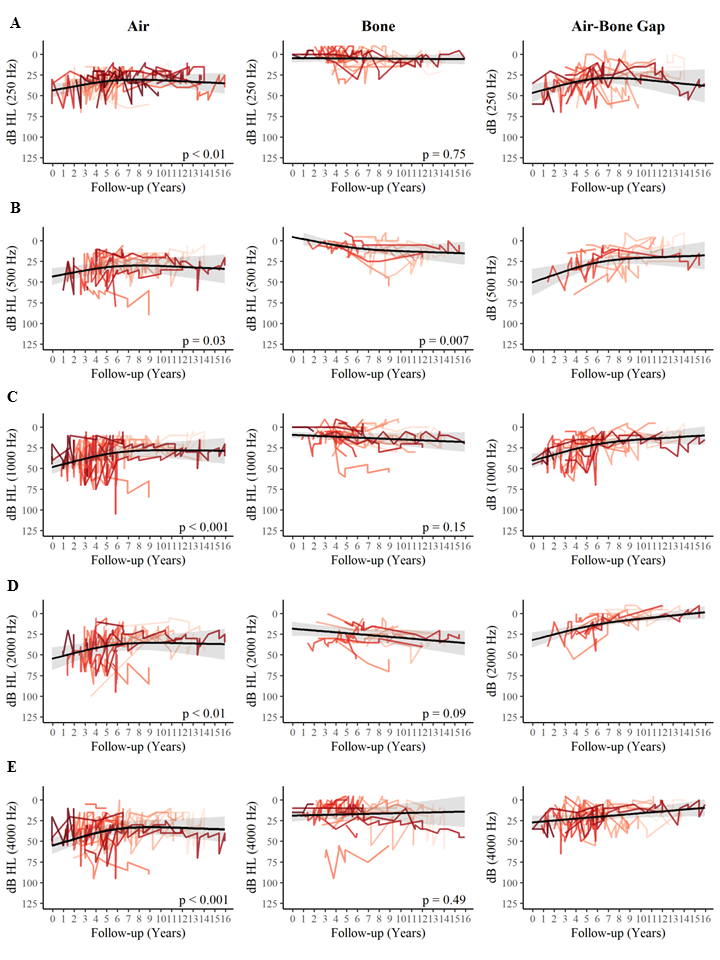

Supplement: Supplementary file 1 — Supplementary Figure S1 Air conduction, bone conduction and air‐bone gap per individual patient over time (blue lines) for the different threshold test frequencies (A: 250 Hz, B: 500 Hz, C: 1000 Hz, D: 2000 Hz, E: 4000 Hz). The black line depicts the average course over time for a patient with normal enzyme activity levels after HCT and mean age at transplantation (1.22 years). [file JIMD-43-1279-s001.tif]
